# Impact of Various Drying Protocols on the Bond Strength of AH Plus Sealer to Dentin Walls Using the Vertical Condensation Technique: An In Vitro Study

**DOI:** 10.7759/cureus.108529

**Published:** 2026-05-08

**Authors:** Amira Arar, Helen R Ayoubi, Yasser Alsayed Tolibah

**Affiliations:** 1 Department of Endodontics and Operative Dentistry, Damascus University, Damascus, SYR; 2 Department of Endodontics and Operative Dentistry, Faculty of Dental Medicine, Damascus University, Damascus, SYR; 3 Department of Pediatric Dentistry, Damascus University, Damascus, SYR

**Keywords:** 70% isopropanol, 95% ethanol, ah plus sealer, paper points, push-out strength

## Abstract

Background

Moisture control within the root canal prior to obturation is a key factor influencing the bond strength of sealers to dentinal walls. Traditionally, paper points have been used to dry root canals, however, this method may not completely eliminate residual moisture, potentially compromising the adhesion of sealers. Recently, the use of chemical drying agents such as ethanol and isopropanol has been suggested to improve dentin dehydration and enhance sealer penetration into dentinal tubules.

Objectives

The main objective of this study is to evaluate the effects of isopropanol alcohol, ethanol alcohol, and paper points as drying protocols on the bond strength of root canal obturation with gutta-percha and AH Plus sealer, using the warm vertical condensation (WVC) technique, to the canal dentinal walls.

Materials and methods

Thirty single-rooted human permanent teeth were decoronated to a length of 15 mm. The roots were randomly divided into three groups (n = 10 for each group) according to the drying protocol: (Group 1 (G1): paper points, Group 2 (G2): 70% isopropanol, and Group 3 (G3): 95% ethanol). Thirty roots were prepared using ProTaper Universal rotary instruments. In G1, the canals were blot dried with paper points until the last one appeared dry; in G2, the canals were dried with paper points followed by dehydration with 70% isopropanol; and in G3, the canals were dried with paper points followed by dehydration with 95% ethanol. After drying, the canals were obturated with AH Plus sealer and gutta-percha using the WVC technique. Then, each root was sectioned into three slices (coronal, middle, and apical thirds) with 2-mm-thick sections using a diamond disc. The push-out strength between the sealer and dentin wall was tested for each slice using a universal testing machine at a crosshead speed of 1 mm/min, and failure modes were examined under a stereomicroscope at 40× magnification. The data were statistically analysed using one-way analysis of variance (ANOVA) and Bonferroni tests. The significance level was set at 0.05.

Results

The three experimental groups demonstrated significant differences (p < 0.05). G2 exhibited significantly higher bond strength than G3 (p < 0.05). In contrast, G1 showed the lowest push-out strength.

Regarding failure modes, statistically significant differences were observed among the groups (p < 0.05). At different root levels, significant differences were also noted (p < 0.05). Specifically, in the coronal third, the majority of failures (80%) in G2 and G3 were cohesive failures, whereas G1 predominantly exhibited mixed failures, followed by a lower frequency of adhesive failures.

Conclusions

Drying with 70% isopropanol and 95% ethanol enhanced the bond strength between AH Plus sealer and dentinal tubules more effectively than the conventional paper point drying method when using the WVC technique, with 70% isopropanol being superior to the other studied groups.

## Introduction

Advancements in obturation techniques, such as warm vertical compaction, have significantly improved the clinician’s ability to achieve a dense, homogeneous fill and enhance the flow of obturation materials into lateral canals and complex anatomical spaces that would otherwise remain unfilled using traditional cold lateral compaction. Numerous studies have demonstrated that three-dimensional obturation reduces microleakage and improves the long-term prognosis of endodontically treated teeth [1].

Epoxy resin-based sealers are frequently used as control materials because of their reduced solubility, long-term dimensional stability, sealing capacity, radiopacity, and adequate microretention to dentin [2]. However, they are highly sensitive to moisture. Excess residual irrigants or water can interfere with polymerisation and reduce bond strength, while excessive drying can collapse dentinal collagen fibrils, limiting sealer penetration into dentinal tubules [3]. Although paper points are the most commonly used method for canal drying in clinical practice, moisture may persist in irregular canal regions and lateral canals even after their use [4]. Since no standardised protocol exists to achieve ideal residual moisture, several chemicals, including alcohol, have been evaluated for their potential to improve dentinal wettability [4].

Ethanol and isopropanol are widely used adjuncts in endodontic practice due to their strong ability to displace residual moisture from dentinal surfaces. Ethanol, particularly at high concentrations such as 95%, and isopropanol, commonly used at 70%, act by reducing surface tension, increasing dentinal surface energy, and lowering the contact angle, thereby improving dentinal wettability and facilitating better sealer penetration and adhesion [5].

Despite the availability of laboratory studies evaluating the effects of ethanol [6] and isopropanol [7] on dentin adhesion and AH Plus performance, no investigation has simultaneously compared these protocols or integrated them with the warm vertical compaction technique using AH Plus. This gap highlights the need for comprehensive studies addressing the combined impact of different drying methods within clinically relevant obturation protocols.

Therefore, this study aimed to evaluate and compare the push-out bond strength and failure modes of the epoxy-based sealer AH Plus, used in warm vertical compaction, after three distinct canal-drying protocols: paper points, 70% isopropanol, and 95% ethanol.

The null hypothesis of this study was that there is no significant difference in mean bond strength across drying protocols and root canal thirds. This article was previously posted on Research Square as a preprint on January 19, 2026 [8].

## Materials and methods

Ethical approval and settings

This study was conducted with ethical approval from the Local Ethics Committee of Damascus University, Damascus, Syria (DN-301225-621). This study was conducted in accordance with the Declaration of Helsinki and followed the CRIS (Checklist for Reporting In-vitro Studies) guidelines [9].

Study design and tooth selection

This experimental study was conducted at the Faculty of Dental Medicine, Damascus University, Department of Endodontics and Operative Dentistry, from June 2024 to August 2025.

Sample size calculation

G*Power 3.1 software (Heinrich-Heine-Universität Düsseldorf, Düsseldorf, Germany) was used to calculate the sample size based on a study by Wang et al. that employed a similar methodology [10]. The power was estimated to be 90%, the probability of Type I error (α) was set at 0.05, and the effect size (f) was 0.53, resulting in a total sample size of 30 teeth.

Sample selection

Thirty human permanent mandibular premolars were used. The premolars were freshly extracted for orthodontic reasons. After obtaining informed consent from the patients to include the extracted teeth in the study, this was done within the approval obtained from the university to conduct the research. The soft tissue residue on the extracted teeth was removed, and the teeth were stored in water at 4°C and used within one month of extraction [11]. Only premolars classified as 1TN1, according to the Ahmed et al. classification [12], were included in the study. Premolars with an open apex, resorption, or curved canals, and teeth that were previously endodontically treated were excluded. Additionally, mesio-distal digital radiographs were used to evaluate the root canal anatomy of all premolars [13], and dentine thickness was measured to exclude teeth with less than 1 mm of dentine thickness. Then, all teeth were examined under a stereomicroscope (Meiji Techno, Saitama, Japan) at 20× magnification to exclude previously cracked or fractured teeth.

Sample preparation

First, the specimens were decoronated by transversely sectioning the roots at 15 mm using a double-faced diamond disc (Hager & Meisinger GmbH, Neuss, Germany) in a low-speed straight handpiece with air/water spray coolant (Being Foshan Medical Equipment Co. Ltd., Foshan, China).

Patency was confirmed with a #10 K-file (MANI, Utsunomiya, Japan), and the root canals were enlarged using ProTaper Universal rotary instruments (Dentsply Sirona, Bensheim, Germany) to size F5, and the working length (WL) was established 1 mm from the apical foramen. All root canal preparations were performed by a single experienced endodontist (A.A.) to minimise inter-operator variability. The root canals were irrigated with 2 mL of 5.25% sodium hypochlorite between each instrument using a syringe with a 30-G side-vented needle (DiaDent, Seoul, South Korea), placed 1 mm short of the WL. After preparation, the canals were irrigated with 2 mL of 17% EDTA (Meta Biomed Co., Ltd., Chungcheongbuk-do, South Korea) for one minute, followed by a final rinse with 5 mL of distilled water. Then, the appropriate master gutta-percha (GP) cones (Meta Biomed Co., Ltd.) were selected for each canal by ensuring that the cone reached the full WL with slight resistance upon withdrawal (tug-back).

The 30 samples were randomly allocated (using a simple computer-generated randomisation) into three experimental groups (n = 10 per group) according to the drying protocol. Group 1: The canals were blot-dried using paper points (Meta Biomed Co., Ltd.) until the last point was visually confirmed to be completely dry [10]. Group 2: After removal of excess distilled water with paper points until complete dryness of the last point was confirmed visually, the root canals were irrigated with 10 mL of 70% isopropanol (OQEMA GmbH, Mönchengladbach, Germany) using a syringe with a side-vented needle (DiaDent) carried to the WL. After being left in the canal for 10 seconds, the isopropanol was removed with paper points [10]. Group 3: After removal of excess distilled water with paper points until complete dryness of the last point was confirmed visually, the root canals were irrigated with 10 mL of 95% ethanol (Chem-Lab NV, now AnalytiChem Belgium NV, Tessenderlo, Belgium) using a syringe with a 30-G side-vented needle (DiaDent) carried to the WL. After being left in the canal for 10 seconds, the ethanol was removed with paper points [10].

The samples in all groups were obturated using a gutta-percha cone and AH Plus sealer (Dentsply Sirona, Bensheim, Germany) using the warm vertical condensation (WVC) technique. The AH Plus sealer was applied using the master cone coating technique, in which the sealer was evenly coated onto the master gutta-percha cone before insertion into the prepared root canal. After placement of the AH Plus, 0.5 mm of the master cone was clipped, and the cone was inserted, with the fastback tip (Fast Fill device, Eighteeth, Changzhou, China) advanced in a single motion to the predetermined depth. Following insertion, the tip was held in place for 15 seconds to cool, after which a one-second heat burst was applied before withdrawing the tip. This procedure was repeated twice: once in the middle third and again in the coronal third of the canal. Excess gutta-percha at the canal orifice was then removed and compacted using a 0.6 mm (No. ½) hand plugger (Dentsply, Tulsa, OK, USA). Finally, the canal orifice was sealed with a temporary filling (Ghimas, Bologna, Italy). All samples were then stored in an incubator (Binder, Tuttlingen, Germany) at 37°C and 100% humidity for seven days to allow complete setting of the sealers [10].

Push-out strength test

**Figure 1:**
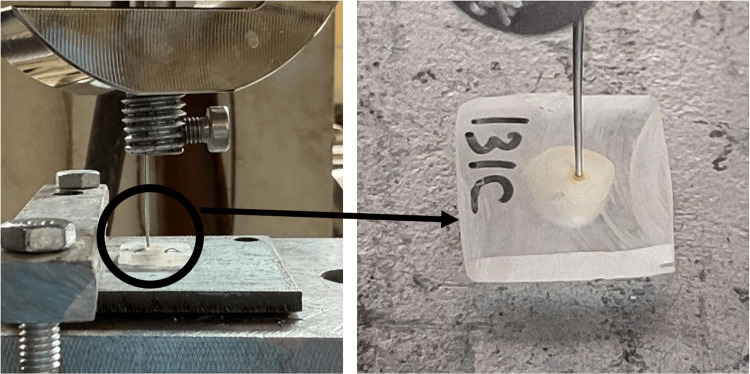
Universal testing machine for the push-out test; the shaft was placed over the tooth slice.

The prepared roots were embedded in a resin block before sectioning to provide stability and ensure standardised slices for the push-out bond strength test. Three slices with 2-mm thickness (one from the apical third, one from the middle third, and one from the coronal third) were cut at intervals of 3, 5, and 9 mm from the apical to the coronal third (30 slices per group) using a low-speed fan-shaped diamond disc with a thickness of 0.25 mm at a rotational speed of 25,000 cycles per minute under copious water cooling at three levels (Hager & Meisinger GmbH, Neuss, Germany). The push-out test was performed using a Universal Testing Machine (Testometric Co. Ltd., Rochdale, UK) at a crosshead speed of 1 mm/min. Shafts with tip diameters of 0.4 mm, 0.8 mm, and 1.0 mm were used for the apical, middle, and coronal sections, respectively (Figure 1) [14]. 

The apical surface displaying the ink dot was placed facing the punch tip, ensuring that loading forces were introduced from an apical-to-coronal direction, thus avoiding any limitation to material movement. This method ensured that the specimen was aligned accurately and reproducibly, maintained the shaft in a central position, and avoided contact with dentin when the material was pushed and dislodged from the canal wall. The push-out strength at failure was calculated in megapascals (MPa) by dividing the load in newtons (N) by the area of the bond interface [10]: bond area: $\pi (R + r)h$, where π = 3.14; R: the radius of the canal close to the crown; r: the radius of the canal close to the apex. Both measurements were calculated in AutoCAD. h: the height of the slice in millimetres, which was standardised at 2 mm for all samples.

Analysis of failure modes

**Figure 2:**
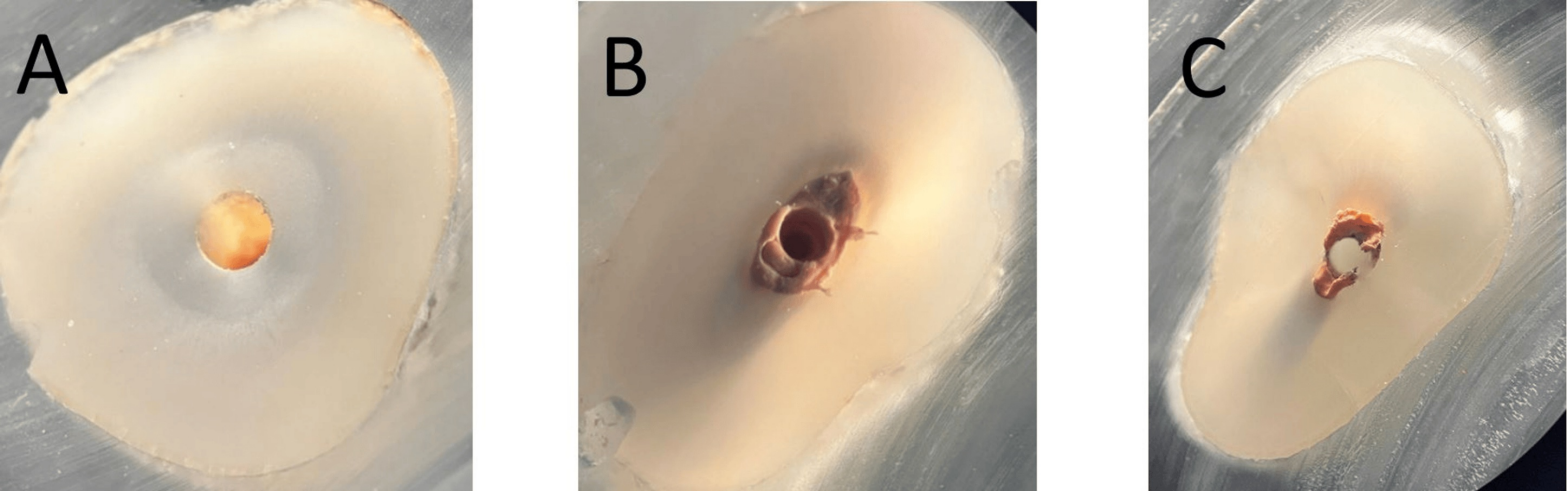
Stereomicroscopic image under 40× magnification: (A) adhesive failure; (B) cohesive failure; (C) mixed failure.

The failure modes were observed under a stereomicroscope at 40× magnification. Failures were classified as adhesive failure (failure occurred between the sealer and the inner root canal wall), cohesive failure (failure occurred inside the sealer), and mixed failure (both failure modes occurred) (Figure 2).

Statistical analysis

Statistical analysis was conducted utilising IBM SPSS Statistics for Windows, Version 24 (Released 2016; IBM Corp., Armonk, NY, USA). Descriptive statistics were obtained for the measured forces (N) and expressed as stress values in megapascals (MPa).

Shapiro-Wilk tests indicated a normal distribution of push-out strength values among the three groups at each third (p > 0.05). Moreover, homogeneity of variance was assessed using the Levene test, which indicated that variances were homogeneous among groups (p > 0.05); hence, intergroup and intragroup comparisons of push-out strength values at each third were performed using one-way analysis of variance (ANOVA), and pairwise comparisons were performed using the Bonferroni test. Data were analysed at a 95% confidence interval, and the significance level was set at 0.05.

## Results

Push-out strength

There was a significant difference in push-out strength between the three groups (p < 0.01). The push-out bond strength values varied significantly across the different drying protocols and root canal sections (coronal, middle, and apical). Specimens dried with 70% isopropanol exhibited the highest bond strength values in all thirds, with mean ± standard deviation (SD) values of 5.96 ± 0.53 MPa, 5.45 ± 0.31 MPa, and 4.74 ± 0.32 MPa in the coronal, middle, and apical thirds, respectively. Similarly, 95% ethanol enhanced bond strength compared to paper point drying, yielding 5.14 ± 0.31 MPa, 4.45 ± 0.42 MPa, and 3.83 ± 0.36 MPa in the coronal, middle, and apical thirds, respectively. In contrast, the paper point protocol resulted in the lowest bond strength values across all thirds (3.65 ± 0.19 MPa, 3.10 ± 0.30 MPa, and 2.61 ± 0.49 MPa, respectively). A consistent trend of decreasing bond strength from the coronal to the apical third was observed in each protocol (Table 1).

**Table 1:** Descriptive statistics of push-out bond strength (MPa) values. Descriptive statistics of push-out bond strength (MPa) values in the studied sample for AH Plus sealer across different canal regions (coronal, middle, and apical) under three drying protocols - paper point, 70% isopropyl alcohol, and 95% ethanol - are presented as mean ± standard deviation. * denotes Mean; t denotes SD.

Drying protocol	Coronal	Middle	Apical
Paper point	^*^3.65 ± ^t^0.19	^*^3.10 ± ^t^0.30	^*^2.61 ± ^t^0.49
70% isopropanol	^*^5.96 ± ^t^0.53	^*^5.45 ± ^t^0.31	^*^4.74 ± ^t^0.32
95% ethanol	^*^5.14 ± ^t^0.31	^*^4.45 ± ^t^0.42	^*^3.83 ± ^t^0.36

Failure mode

Cohesive failure was the predominant mode across all root thirds, particularly in specimens dried with 70% isopropanol and 95% ethanol. In the coronal third, cohesive failures accounted for 80% of samples in both alcohol-based protocols, compared with 60% with paper-point drying. Mixed failures were more frequent with paper point (30%) than with isopropanol or ethanol (20%). Adhesive failures were observed only in the coronal third during paper-point drying (10%).

In the middle third, cohesive failures ranged from 60% to 70%, with ethanol showing the highest proportion of mixed failures (40%). No adhesive failures were detected in this third under any protocol.

In the apical third, paper-point drying resulted in the lowest cohesive failure rate (20%) and the highest mixed failure rate (60%), with 20% adhesive failures. In contrast, isopropanol and ethanol protocols yielded higher cohesive failure rates (70% and 60%, respectively) and no adhesive failures (Table 2).

**Table 2:** Failure mode distribution at each root third (coronal, middle, and apical) of AH Plus sealer and vertical compaction.

Root sections	Drying protocol	Failure mode			All sections
		Cohesive (%)	Adhesive (%)	Mix (%)	
Coronal	Paper point	6 (60%)	10 (10%)	3 (30%)	10
	70% isopropanol	8 (80%)	0 (0%)	2 (20%)	10
	95% ethanol	8 (80%)	0 (0%)	2 (20%)	10
Middle	Paper point	7 (70%)	0 (0%)	3 (30%)	10
	70% isopropanol	7 (70%)	0 (0%)	3 (30%)	10
	95% ethanol	6 (60%)	0 (0%)	4 (40%)	10
Apical	Paper point	2 (20%)	2 (20%)	6 (60%)	10
	70% isopropanol	7 (70%)	0 (0%)	3 (30%)	10
	95% ethanol	6 (60%)	0 (0%)	4 (40%)	10

## Discussion

It should be noted that laboratory tests cannot predict the clinical behaviour of materials; since teeth are located in the alveolus, many other factors, such as the periodontal ligament and the temperature of the oral cavity, can influence the properties of an endodontic sealer [15]. Although bond strength testing may not be a reliable predictor of the clinical behaviour of sealers [15], push-out bond strength testing has been considered suitable for comparing bond strength and evaluating failure patterns of different materials or techniques under controlled conditions, thereby minimising bias [15].

Root canal filling is accomplished through the combination of a solid core material - commonly gutta-percha - with a fluid endodontic sealer [16]. The solid material serves as the central core, while the sealer functions to cement or fix this core within the canal system [2]. In doing so, the sealer fills irregularities, voids, and extensions such as lateral or accessory canals that cannot be completely occupied by the core material alone [16].

Push-out strength is commonly employed as an indicator of the adhesion between obturation materials and root canal walls due to its methodological simplicity and reproducibility [17]. This parameter reflects a composite measure encompassing frictional resistance at the material-dentin interface, intermolecular bonding forces, and chemical adhesion to root dentin [18]. Additionally, push-out strength is influenced by factors such as frictional forces [19], the polymerisation shrinkage factor (C-factor) [20,21], and different root canal treatment protocols [22].

It is suggested that the push-out test offers a more accurate evaluation of adhesive strength than the traditional shear test [23]. This is primarily because the push-out test induces fracture parallel to the dentin-sealer interface, thereby representing a true shear stress condition in specimens with parallel-sided geometry [23].

In this study, the drying protocols exerted distinct influences on the push-out bond strength and the penetration of resin-based sealer into dentinal tubules; therefore, the null hypothesis was rejected. Canals dried with 70% isopropanol or 95% ethanol exhibited significantly greater bond strength compared to those dried with paper points. Conversely, canals dried with 70% isopropanol showed the highest overall bond strength. Paper points rely on the principle of direct contact and capillary action to absorb and adsorb water. However, due to the complex anatomy of the root canal system, some residual moisture may remain, forming a non-displaceable physical barrier that can hinder complete endodontic penetration [24]. However, Nagas et al. found that moisture remained in the irregular regions of the canal and in lateral canals after drying with paper points. This residual moisture may hinder complete sealer penetration and compromise the bond strength to dentin. Such findings highlight the limitations of paper-point drying alone [4].

This finding is consistent with previous studies comparing paper-point and 70% isopropyl alcohol protocols [24], which showed improved bond strength due to better moisture control. Similarly, research on ethanol treatment reported that dentin exposure to 70%-100% ethanol increased surface free energy and wettability, reducing the contact angle of AH Plus and enhancing sealer-dentin interaction [7].

Several factors may explain why isopropanol provides stronger sealer-dentin adhesion compared to ethanol. Considering the hydrophilic propensity of resin-based sealers, it may be speculated that isopropyl alcohol (C₃H₇OH), which has lower polarity than ethanol (C₂H₅OH), promotes less removal of water from dentinal tubules, thereby enhancing dentin wettability and increasing the degree of conversion of the sealers [25]. In contrast, ethanol at high concentrations can excessively dehydrate the collagen network within dentin, leading to partial collapse of its structure [6]. Unlike ethanol, isopropanol leaves a small amount of residual moisture within the dentin collagen matrix, helping preserve its structural flexibility and allowing improved sealer penetration [8]. Moreover, resin-based sealers often require a slightly moist environment to optimise polymerisation. Isopropanol provides a balanced condition, whereas ethanol may leave a surface that is overly dry, reducing the degree of conversion. Additionally, due to its viscosity and physical properties, isopropanol may facilitate deeper penetration into dentinal tubules compared to ethanol, thereby enhancing mechanical contact between the sealer and dentin [26].

Previous studies [4] have shown that complete drying of root canal dentin with high-concentration ethanol removes most of the residual moisture from dentinal tubules. While this creates a very clean surface, it may eliminate the minimal moisture necessary for optimal AH Plus adhesion. Consequently, moderate moisture levels (moist dentin) were found to provide higher sealer bond strength than overdried or fully wet conditions, highlighting the importance of controlling canal moisture for effective resin-dentin interaction [4].

Ethanol evaporates rapidly, leading to excessive dehydration and collagen shrinkage, whereas isopropanol’s slower evaporation rate maintains a more stable dentinal surface for sealer adhesion [26].

The differences in push-out bond strength among the coronal, middle, and apical thirds can be explained by several factors. The coronal third generally exhibits the highest values due to greater dentinal tubule density, more effective irrigant penetration, and easier smear layer removal [27,28]. In addition, condensation of the sealer and gutta-percha is more efficient in this region because of the wider canal diameter and better accessibility [29]. The middle third showed moderate bond strength, reflecting a gradual decrease in tubule density and slightly reduced condensation efficiency, although irrigants and sealers can still penetrate adequately. Conversely, the apical third demonstrates the lowest bond strength, which may be attributed to reduced tubule density, complex apical anatomy, limited irrigant penetration, residual smear layer, and less effective condensation due to restricted access and narrow canal dimensions [8].

Furthermore, instrumentation pressure during mechanical preparation may induce subtle alterations in the canal walls, with a more pronounced effect in the coronal and middle thirds compared to the apical third [30]. These changes influence the surface characteristics available for bonding and may partly explain the higher bond strength observed coronally and in the middle segment. In addition, sealer distribution tends to be more uniform in the coronal and middle thirds, whereas in the apical third, the narrower canal diameter and complex anatomy often lead to uneven accumulation of the sealer [30]. Together, these factors contribute to the differences in push-out bond strength among the three canal regions.

Canals dried with 70% isopropanol or 95% ethanol showed no adhesive failure along the sealer-dentin interface, a result that may be partly attributed to the bonding forces established between the sealer and the dentinal surface. In contrast, adhesive failure was observed when canals were dried using paper points alone, highlighting the influence of the drying protocol on the integrity of the sealer-dentin interface [4].

When canals were dried with 70% isopropanol, ethanol 95% showed a relatively high cohesive failure mode, suggesting that alcohol allows for stronger interaction with the canal dentin wall. Mixed failure was observed in 60% of the apical third specimens in the paper-points group, whereas 20% exhibited adhesive failure. This pattern may be partly explained by the complex apical anatomy, including the presence of secondary canals, which can hinder sealer solidification and limit its interaction with the canal walls [10]. Notably, the incidence of mixed failure decreased to 40% in the apical third when canals were dried using ethanol and further declined to 30% when isopropanol was applied. Under these drying conditions, cohesive failure became more predominant, suggesting improved sealer adaptation and stronger interaction with the dentinal walls.

Although the present findings demonstrated superior bond strength with 70% isopropanol, previous studies have reported variable outcomes regarding alcohol-based drying protocols. Nagas et al. demonstrated that excessive dentin dehydration may adversely affect resin sealer adhesion by altering collagen integrity, whereas moderate residual moisture may favour AH Plus interaction with dentin [4]. Furthermore, Sarrafan et al. observed that the influence of drying protocols differed among endodontic sealers, suggesting that material composition and hydrophilicity may significantly affect bonding behaviour [31]. These discrepancies may be attributed to differences in obturation techniques, irrigation regimens, alcohol concentration, canal anatomy, and testing methodologies.

To validate and extend these findings beyond the in vitro setting, future clinical trials that closely replicate the current experimental design are strongly recommended. Such investigations would enable the translation of laboratory outcomes into clinical practice, thereby substantiating the clinical relevance of isopropanol drying as a final step in root canal preparation and confirming its potential to enhance the long-term success of obturation.

One of the limitations of this study is its in vitro nature, as it did not focus on the overall quality of the filling achieved using a final irrigation protocol containing isopropyl alcohol or ethanol, nor on the potential adverse effects of these compounds on periapical tissues. Future studies should address these aspects. Moreover, although multiple slices were obtained from each tooth to assess different root canal thirds, it should be recognised that these measurements may not be entirely independent, because they originated from the same specimen. Future studies may consider hierarchical or mixed-effects statistical models to better account for within-tooth correlations.

## Conclusions

Within the limitations of this in vitro study, drying the canals with 70% isopropyl alcohol yielded the highest bond strength between AH Plus sealer and dentinal tubules when using the WVC technique. Although 95% ethanol also improved bonding, its effect remained inferior to that of 70% isopropanol, while the paper-points method consistently produced the lowest values. These findings highlight the clear superiority of alcohol-based drying protocols over conventional drying approaches.
